# Exploring the metabolic burden of surfactin biosynthesis and the metabolic costs of *srfA* operon expression in *Bacillus subtilis*

**DOI:** 10.1186/s12934-026-03047-1

**Published:** 2026-06-19

**Authors:** Elvio Henrique Benatto Perino, Eric Hiller, Maliheh Vahidinasab, Sandra Moneta, Bahar Abrishamchi, Kowsala Nagendran, Philipp Hubel, Jens Pfannstiel, Rudolf Hausmann

**Affiliations:** 1 Department of Bioprocess Engineering (150k), Institute of Food Science and Biotechnology, University of Hohenheim, Fruwirthstrasse 12, 70599 Stuttgart, Germany; 2 Core Facility Hohenheim, Mass Spectrometry Unit, University of Hohenheim, August-von-Hartmann-Str. 3, 70599 Stuttgart, Germany

**Keywords:** Surfactin, Metabolic burden, *Bacillus subtilis*, NRPS, Kinetic modelling, Lipopeptides

## Abstract

The biosynthesis of surfactin, a potent lipopeptide biosurfactant produced by *Bacillus subtilis*, imposes a significant metabolic burden on the host organism. Understanding the metabolic costs associated with surfactin production, specifically the ATP demand and precursor diversion resulting from the expression of the large *srfAA-AD* operon (*srfA* operon), is crucial for optimizing production strains. In this study, the metabolic burden and growth impacts associated with surfactin biosynthesis in *B. subtilis* were quantitatively evaluated by comparing a reference surfactin-producing strain (BMV9) with two mutant strains: BMV12, which lacks the *srfA* operon, and BMV33, which retains the *srfA* operon but lacks the *sfp* gene. Our analysis included theoretical calculations of ATP and NADPH + H^+^ requirements for *de novo* surfactin synthesis, and we measured growth behavior and carbon and nitrogen source consumption. Results indicated that surfactin production significantly reduces biomass yield (*Y*_*X/S*_) and specific growth rates due to the metabolic costs of expressing the non-ribosomal peptide synthetase (NRPS) and the diversion of key precursors. Notably, BMV12 exhibited higher growth rates compared to the surfactin-producing strain. Proteome analyses further revealed differential protein abundance in non-surfactin-producing strains, indicating altered metabolic pathways that may relieve the metabolic burden associated with surfactin synthesis. These findings highlight the complex trade-offs between secondary metabolite production and cellular growth, providing a foundation for metabolic engineering strategies aimed at optimizing surfactin yields while minimizing metabolic costs.

**Supplementary Information:**

The online version contains supplementary material available at 10.1186/s12934-026-03047-1.

## Introduction

The production of secondary metabolites, such as surfactin, by *Bacillus subtilis* represents a significant area of interest due to their application potential in biotechnology, bioremediation, agriculture and industry. Surfactin is a cyclic lipopeptide composed of seven amino acids linked to a β-hydroxy fatty acid. Structurally, all members of the surfactin family consist of a cyclic peptide composed of seven amino acids linked to a fatty acid chain, which can be linear, iso, or anteiso, with branches containing 12 to 17 carbon atoms, often identified as 3-hydroxy-13-methyltetradecanoic acid [1]. The most common amino acid sequence in the surfactin cyclic peptide consists of l-glutamate, l-leucine, d-leucine, l-valine, l-aspartate, d-leucine, and l-leucine. To date, more than 40 variants of surfactin have been identified [2]. It is produced by various *Bacillus* species and is known for its strong biosurfactant activity. Since its discovery by Arima et al. [3] numerous research studies have highlighted the significant biosurfactant activities of surfactin and its application potential in many industrial sectors such as cosmetics, pharmaceuticals, as well as food [2, 4–6].

Surfactin is synthesized non-ribosomally by a multi-modular enzyme complex encoded by the *srfA* operon (26,073 bp). Its production is further dependent on the presence of the *sfp* gene, which encodes a 4′-phosphopantetheinyl transferase essential for a post-translational activation of the surfactin-forming non-ribosomal peptide synthetase (NRPS) [7–10]. Accordingly, *Bacillus* strains with an unfunctional *sfp* gene, such as the laboratory model strain *B. subtilis* 168 and the sporulation-deficient strain 3NA, cannot produce surfactin or other lipopeptides like plipastatin [11, 12]. Therefore, to convert these strains into lipopeptide-producing strains, the *sfp* pseudogene must first be repaired [6, 13].

Wild-type *Bacillus* species usually produce up to a gram per liter of surfactin under optimal laboratory conditions [14]. However, the production titer of wild-type *Bacillus* species does not meet the requirements for industrial use. To address this issue, genetic modification of producer strains and bioprocess engineering techniques can boost the production of lipopeptides [6, 15]. Consequently, the highest reported amount of surfactin production to date, reaching over 45 g/L, was achieved by *B. subtilis* BMV9 in a high cell-density fed-batch bioreactor cultivation [16]. However, the regulation and mechanisms underlying the biosynthesis of *Bacillus* lipopeptides are not yet fully understood. Several studies continue to be conducted to explore these areas and identify ways to improve production efficiency [17, 18].

Surfactin production requires a substantial supply of ATP and NADPH, and the translation and assembly of the NRPS complex represents a major commitment of cellular resources. Although several studies have investigated precursor supply pathways for branched-chain amino acids and β-hydroxy fatty acids, no quantitative assessment has been made of the metabolic costs directly linked to *srfA* operon expression, NRPS translation, and surfactin formation in *B. subtilis*. Despite advances in metabolic and bioprocess engineering that have enabled high surfactin titers, the physiological burden associated with the expression of the *srfA* operon and the biosynthesis of surfactin itself remains insufficiently understood. The biosynthesis of surfactin is a metabolically resource intensive process that imposes a considerable burden on the host organism, *B. subtilis*. Understanding the metabolic implications of surfactin production, including the resource demands and the diversion of key precursors, is crucial for optimizing production processes and improving yields.

An important area of research to increase surfactin production is to improve the metabolic pathways that supply β-hydroxy fatty acids and the amino acids involved in surfactin structure [19, 20]. However, there are no studies on estimation of the metabolic costs associated with *srfA* operon expression and metabolic burden needed for the biosynthesis of surfactin by the NRPS in *B. subtilis*.

The metabolic implications of surfactin biosynthesis were examined using three isogenic *B. subtilis* strains that allow the effects of operon expression, NRPS translation and surfactin production to be distinguished. BMV9, a surfactin-producing strain with an intact *srfA* operon and *sfp* gene, was compared with BMV12, which lacks the *srfA* operon entirely, and BMV33, which retains the entire operon but lacks *sfp* and therefore expresses the NRPS without synthesising surfactin. This set of strains enables a systematic analysis of the metabolic and physiological consequences associated with the operon being absent, present but inactive, or fully functional. Because each genetic configuration imposes a different metabolic burden on the cell, differences in growth behaviour are expected to reflect the costs associated with NRPS expression and surfactin biosynthesis.

Despite significant advances in strain and process engineering, the physiological costs associated with surfactin biosynthesis remain poorly understood. From a microbial cell factory perspective, quantifying metabolic burden is particularly important because the expression of large biosynthetic gene clusters and the synthesis of complex secondary metabolites can divert energy, reducing equivalents, and precursor metabolites away from biomass formation and other cellular functions. A better understanding of these costs is therefore essential for the rational design of improved production strains and cultivation strategies that balance cellular fitness and product formation.

The aim of this study was to quantify the energetic requirements for *de novo* surfactin synthesis, as previously described by Henkel et al. [21] for rhamnolipid production, and to compare the growth characteristics and substrate utilisation of the three strains. In addition, kinetic modelling was applied to evaluate how *srfA* operon expression influences growth parameters and metabolic burden, including maximum growth rates, biomass formation, glucose consumption, surfactin formation, and biomass-to-substrate yields. Proteomic analysis was performed as an independent systems-level approach to investigate global cellular responses associated with NRPS expression and Sfp-dependent surfactin biosynthesis and to identify metabolic adaptations linked to the observed growth differences. Through this integrated approach, the study provides new insight into the metabolic constraints associated with surfactin biosynthesis and establishes a foundation for optimising the performance of future surfactin-producing strains.

## Materials and methods

### Strain construction

**Fig. 1:**
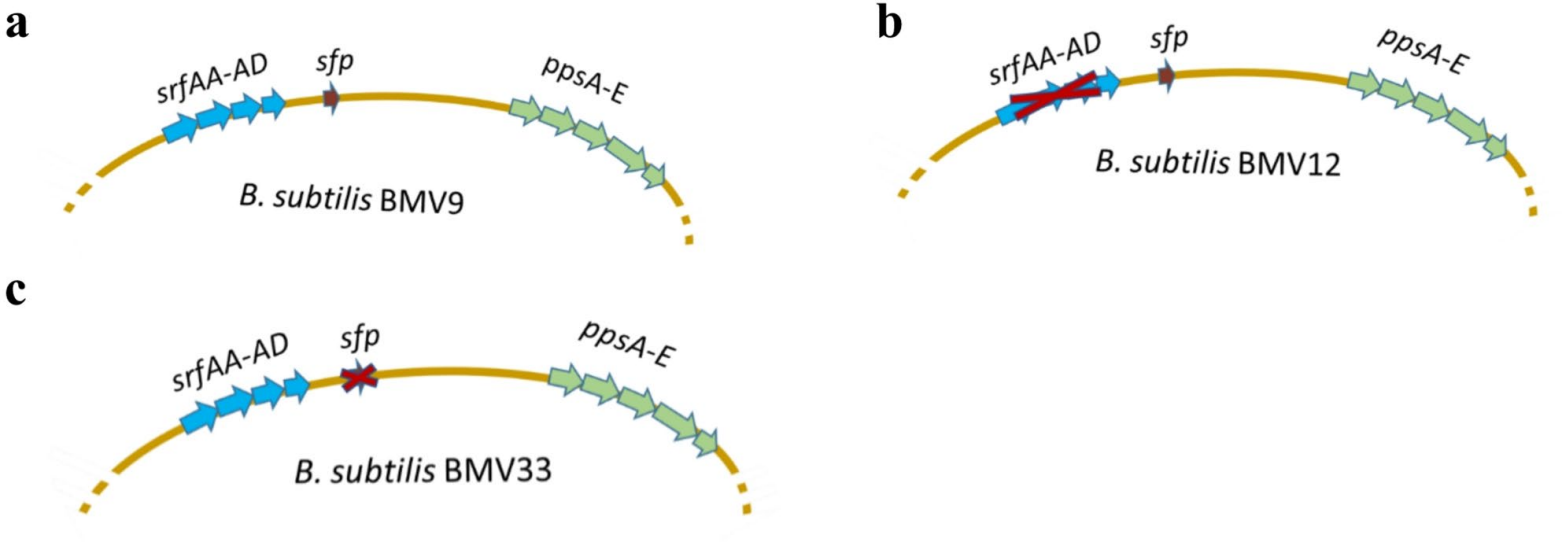
Schematic illustration showing the main features of the *B. subtilis* strains used in this study. **a** *B. subtilis* BMV9, a surfactin-producing strain harboring the *srfAA-AD* operon and *sfp* gene; **b** *B. subtilis* BMV12, a non-surfactin-producing strain due to the deletion of the *srfAA-AD* operon; **c** *B. subtilis* BMV33, a non-surfactin-producing strain due to the deletion of the *sfp* gene

The main genotype features of the three bacterial strains used in this study are shown in Fig. 1. The strain *B. subtilis* BMV9 is a *sfp*^+^ derivative of the sporulation deficient strain.

*B. subtilis* 3NA [12, 22] and was used as a reference strain for surfactin production and the associated cell growth and glucose consumption over the cultivation. Strain BMV12 is derived from BMV9, featuring a complete deletion of the *srfA* operon; its construction method has been described previously [12]. BMV33 also originates from BMV9 and shows a deletion of the *sfp* gene. For construction of BMV33, the *sfp* knockout locus from the *Bacillus* knockout strain BKE03570 was transformed into the BMV9 strain [23].

All mutated chromosomal regions were amplified using Phusion High-Fidelity DNA Polymerase (New England BioLabs, Frankfurt am Main, Germany) and were purified using the QIAquick PCR and Gel Cleanup Kit (Qiagen, Hilden, Germany), according to the manufacturers’ instructions. PCR fragments were sequenced by Eurofins Genomics Germany GmbH (Ebersberg, Germany). A list of oligonucleotides used in this study is provided in supplementary file 1.

### Calculation of the theoretical molecular burden for *de novo* surfactin synthesis in *B. subtilis*

To estimate the theoretical molecular burden for *de novo* synthesis of surfactin, several factors have been considered. Firstly, the energy requirement in the form of ATP and reducing power from NADPH + H^+^ for biosynthesis of the precursors of surfactin has been calculated. These precursors include l-aspartate, l-glutamate, l-valine and four l-leucine that form the peptide ring of surfactin, as well as the 3-hydroxy fatty acid as a dominant fatty acid in the structure of surfactin. These demands were summed up with the ATP required for activating amino acids by NRPS enzyme complex.

### Media and shake flask cultivation

Luria-Bertani (LB) medium was used for preparation of pre-cultures. An inoculum of 10 µL of glycerol stock of bacterial strains was used into 10 mL LB medium and was incubated overnight at 37 °C and 120 rpm. The pre-culture was used to inoculate 100 mL mineral salt medium with an initial optical density (OD_600_) of 0.1 in a 1 L shake flask. The composition of the mineral salt medium is the following: 4.0 × 10^− 6^ M Na_2_EDTA × 2 H_2_O, 7.0 × 10^− 6^ M CaCl_2_, 4.0 × 10^− 6^ M FeSO_4_ × 7 H_2_O, 1.0 × 10^− 6^ M MnSO_4_ × H_2_O, 50 mM (NH_4_)_2_SO_4_, 30 mM KH_2_PO_4_, 40 mM Na_2_HPO_4_ × 2 H_2_O and 8.0 × 10^− 4^ M MgSO_4_ × 7 H_2_O. Glucose was used as the sole carbon source at concentrations of 8 g/L. The experiment was performed in biological triplicates at 37 °C for a cultivation time of 10 h.

### Surfactin and glucose analysis by high-performance thin-layer chromatography (HPTLC)

During the cultivation, samples were taken regularly every hour, and cell-free supernatants were obtained to measure surfactin and glucose concentrations. An appropriately diluted cell-free supernatant was prepared for glucose analysis with an enzymatic assay kit (R-Biopharm AG, Darmstadt, Germany), while 2 mL of the cell-free supernatant was used for surfactin extraction with a chloroform/methanol (2:1, v/v) solvent mixture as described before [24]. Briefly, a three-time extraction of 2 mL cell-free broth with each 2 mL chloroform/methanol 2:1 (v/v) was conducted. The pooled solvent layers obtained after each extraction were evaporated to dryness in a rotary evaporator (RVC2-25 Cdplus, Martin Christ Gefriertrocknungsanlagen GmbH, Osterode am Harz, Germany) at 10 mbar and 40 °C. For HPTLC analysis, samples were resuspended in 2 mL methanol and applied as 6 mm bands on HPTLC silica gel 60 plates from Merck (Darmstadt, Germany). A surfactin standard curve was applied in the range of 30–600 ng/band. The development was conducted using chloroform/methanol/water (65:25:4, v/v/v) over a migration distance of 60 mm. After the development, the plate was scanned at 195 nm to quantify surfactin.

### Proteome analysis

#### Sample preparation

Samples from the late exponential phase of each strain’s cultivation were collected to compare cell differentiation among different strains. Sample of 1 ml were centrifuged at 12,000 g for 10 min. After removing the supernatant, the cell pellet was used for proteome analysis.

Samples were lysed in 4% SDS, 100 mM Tris HCl pH 8.5 for 5 min at 95 °C. Lysates were cleared by centrifugation at 20,000 g, reduced and alkylated in 10mM TCEP, 40 mM CAA for 20 min at 60 °C in the dark and precipitated for 16 h at -20 °C in 80% acetone. Precipitated proteins were pelleted by centrifugation at 20,000 g for 15 min (3 °C). Air dried protein pellets were solubilized in 0.2% SDC, 100 mM Tris HCl pH 8.5 and protein concentrations was determined by a Bradford assay (Roti-Quant, Roth). Lysates were adjusted to a final protein concentration of 1 µg/µl. For protein extraction, 30 µl of the lysate was used by Single-Pot Solid-Phase-enhanced Sample Preparation (SP3; 1:1 Mixture of SpeedBeads™ magnetic carboxylate modified particles 50 mg/ml; Cytiva; CAT No: 45152105050250 and 65152105050250) [25]. Proteins were bound to the magnetic beads by adding ethanol to a final concentration of 70%. Bead bound proteins were washed twice with 70% Ethanol and digested on the beads in 0.2% SDC, 50mM Ammoniumbicarbonat, in a protease to protein ratio of 1:100 Trypsin (Roche) and 1:200 LysC (Walko) respectively (20 h, 37 °C, 800 rpm). Formic acid (FA) was added to the samples to a final concentration of 2%, precipitated SDC was pelleted by centrifugation at 20,000 g for 15 min, peptides in the supernatant was concentrated and desalted on C18 Stage Tips as described by Rappsilber et al. [26] and dried under vacuum. Dried samples were dissolved in 0.1% TFA.

#### LC-MS/MS data analysis

NanoLC-MS/MS experiments were performed on an Ultimate 3000 nano-RSLC (Thermo Fisher Scientific) coupled to an Exploris 480 mass spectrometer (Thermo Fisher Scientific) using a Nanospray-Flex ion source (Thermo Fisher Scientific). Peptides were concentrated and desalted on a trap column (5 mm x 30 μm, Thermo Fisher Scientific) and separated on a 25 cm x 75 μm nanoEase MZ HSS T3 reversed-phase column (100 Å pore size, 1.8 μm particle size, Waters, USA) operated at constant temperature of 35 °C. Peptides were separated at a flow rate of 300 nL/min using a gradient with the following profile: 2% − 8% solvent B in 2 min, 8% − 30% solvent B in 32 min, 30% − 47% solvent B in 13 min, 47% − 96% solvent B in 3 min, isocratic 96% solvent B for 5 min, 96% − 2% solvent B in 5 min and isocratic 2% solvent B for 5 min. The solvents used were 0.1% FA (solvent A) and 0.1% FA in ACN/H2O (80/20, v/v, solvent B). MS spectra (m/z = 300–1400) were detected in the Orbitrap at a resolution of 60,000 (m/z = 200). The maximum injection time (MIT) for MS spectra was set to 50 ms, the automatic gain control (AGC) value was set to 3 × 10E6. Internal calibration of the Orbitrap analyzer was performed using lock-mass ions from ambient air as described in Olsen et al. [27]. The MS was operating in the data-dependent mode selecting the top 30 highest abundant peptide precursor signals for fragmentation (HCD, normalized collision energy of 30). For MS/MS analysis only precursor charge states from 2 to 4 were considered, the monoisotopic precursor selection was set to peptides, and the minimum intensity threshold was set to 2 × 10E5. MS/MS scans were performed in the Orbitrap with a resolution of 150,000, isolation width was set to 1.6 Da. The AGC target was set to 8 × 10E4, a max injection time was set to 40 ms and the first mass was set to 120 m/z. Dynamic exclusion was set to 60 s with a tolerance of 5 ppm.

#### LC-MS/MS data analysis

Raw files were imported into MaxQuant [28] version 2.0.1.0 for protein identification and label-free quantification (LFQ) of proteins. Protein identification in MaxQuant was performed using the database search engine Andromeda [29]. MS spectra and MS/MS spectra were searched against *Bacillus Subtilis* (strain 168) sequence database downloaded from UniProt [30]. Reversed sequences as decoy database and common contaminant sequences were added automatically by MaxQuant. Mass tolerances of 4.5 ppm (parts per million) for MS spectra and 20 ppm for MS/MS spectra were used. Trypsin was specified as enzyme and two missed cleavages were allowed. Carbamidomethylation of cysteines was set as a fixed modification and protein N-terminal acetylation and oxidation were allowed as variable modifications. The ‘match between runs’ feature of MaxQuant was enabled with a match time window of 0.7 min and an alignment time window of 20 min. Peptide false discovery rate (FDR) and protein FDR thresholds were set to 0.01.

MaxQuant output file (protein groups table) was loaded into Perseus version 1.6.14.0 [31]. LFQ values from MaxQuant were log_2_ transformed and matches to contaminant (e.g., keratins, trypsin) and reverse databases identified by MaxQuant were excluded from further analysis. Two samples Welch´s t-test and Volcano plots were performed using Perseus version 1.6.14.0. Only proteins with a minimum of three valid LFQ quantifications in at least one group of replicate experiments (*n* = 3) in the data set were considered for the statistical analysis. Remaining missing values were imputed in R (https://www.r-project.org, version 3.6.2) using the QRILC (Quantile Regression Imputation of Left-Censored data) function in the imputeLCMD package [32–34] with a tune sigma value of one. Significant changes in protein abundance were analysed by a Welch’s t-test (two sided, S0 = 1) and corrected for multiple hypothesis testing using permutation-based FDR statistics (FDR = 0.05, 250 permutations).

### Data evaluation and cultivation parameter

Cell dry weight (CDW) was estimated from OD_600_ measurements using a previously determined conversion factor of 4.31 [35].

The biomass yield (*Y*_*X/S*_), substrate to product conversion yield (*Y*_*P/S*_), product yield (*Y*_P/X_), and productivity of biomass (*q*_*P/X*_) were calculated according to the equations provided before [36].

### Kinetic modelling

#### Modelling platform

The modelling was performed using the mathematical/numerical program MTLAB R2023a (MATLAB, The Math-Works, Natick, MA, USA). The system of differential equations was solved with the implicit multi-step solver “ode15s” from the MATLAB environment (Shampine and Reichelt 1997).

#### Nomenclature

The kinetic model consists of the biomass X, the substrate (glucose) S and the product (surfactin) P as well as the time t. An overview of all model parameters along with their definitions and units is provided in Table 1.

**Table 1:** Overview of all model parameters and variables, along with their units and definitions

Model parameters	Unit	Definition
X	g/L	Biomass concentration
X_0_	g/L	Initial biomass concentration
S	g/L	Substrate concentration
S_0_	g/L	Initial substrate concentration
P	g/L	Product concentration
P_0_	g/L	Initial product concentration
t	h	Process time
µ	1/h	Specific growth rate of biomass X on substrate S
µ_max_	1/h	Maximum specific growth rate of biomass X on substrate S
Ks	g/L	Half-saturation constant substrate S
m	g/(g*h)	Maintenance substrate S
t_lag_	h	Duration of lag phase
Y_X/S_	g/g	Conversion yield of substrate S to biomass X
Y_P/S_	g/g	Conversion yield of substrate S to product P
Y_NRPS/X_	g/g	Production yield of NRPS by biomass X
Y_NRPS/S_	g/g	Conversion yield of substrate S to NRPS
α	molecules/molecule	Formation of molecules P by molecule NRPS
M_P_	g/mol	Molecular weight of product P
M_NRPS_	g/mol	Molecular weight of NRPS
q_P_	g/(g*h)	Production rate of product P
i	-	Strain selection parameter (i = 9, 12 or 33)

### Model setup

#### Initial conditions

The initial values of biomass X_0_, substrate (glucose) S_0_ and product (surfactin) P_0_ were calculated separately for each strain (BMV9, BMV12 and BMV33) from the experimental data at the beginning of the shake flask cultivations.

#### Biomass growth

The growth of biomass X (Eq. 1) was expressed as directly proportional to the existing biomass and to the specific growth rate µ for glucose as the substrate.

1 $$\:\frac{dX}{dt}=\:\mu\:\:\cdot\:X$$

The specific growth rate µ (Eq. 2) was formulated based on Monod kinetics [37]. Here, µ_max_ means the maximum specific growth rate of biomass X on the substrate S and K_S_ represents the half-saturation constant, with a fixed value as reported by Henkel et al., [38].

2 $$\:\mu\:=\:{\mu\:}_{max}\:\cdot\:\:\frac{S}{S+\:{K}_{S}}$$

#### Substrate consumption

The consumption of the substrate (glucose) S was expressed by a differential equation depending on the *B. subtilis* strain. In the case of BMV9 (Eq. 3), the substrate is consumed for biomass growth X as well as for the synthesis of the product of interest (surfactin) P. In addition, substrate is used for the formation of NRPS and for maintenance metabolism, represented by the factor m. Conversion yields for biomass Y_X/S_, for the product Y_P/S_, and for NRPS Y_NRPS/S_ were applied. Furthermore, Y_NRPS/X_ describes the formation of NRPS by the biomass X.

3 $$\:\frac{dS}{dt}=\:-\:\frac{\mu\:}{{Y}_{X/S}}\:\cdot\:X-m\:\cdot\:X\:-\:\frac{\mu\:\:\cdot\:\:{Y}_{NRPS/X}}{{Y}_{NRPS/S}}\:\cdot\:X-\:\frac{1}{{Y}_{P/S}}\:\cdot\:\:\frac{dP}{dt}$$

For BMV12 (Eq. 4), the substrate differential equation is considerably simplified. Since the strain produces neither NRPS nor product (surfactin) P, the substrate (glucose) S is used solely for biomass growth X and for maintenance metabolism m.

4 $$\:\frac{dS}{dt}=\:-\:\frac{\mu\:}{{Y}_{X/S}}\:\cdot\:X-m\cdot\:X$$

BMV33 (Eq. 5) is capable synthesizing NRPS but not the product of interest (surfactin) P. For this reason, the differential equation for substrate (glucose) S consumption is simplified to include only biomass growth X, NRPS formation, and maintenance metabolism m.

5 $$\:\frac{dS}{dt}=\:-\:\frac{\mu\:}{{Y}_{X/S}}\:\cdot\:X-m\:\cdot\:X\:-\:\frac{\mu\:\:\cdot\:\:{Y}_{NRPS/X}}{{Y}_{NRPS/S}}\:\cdot\:X$$

#### Product formation

The formation of the target product (surfactin) P, was calculated to be directly proportional to the production rate *q*_*P*_ multiplied by the biomass X, as described in Eq. 6.

6 $$\:\frac{dP}{dt}=\:{q}_{P}\:\cdot\:X$$

The calculation of the product (surfactin) P production rate *q*_*P*_ was done according to Eq. 7. The parameter α denotes the formation coefficient describing how many molecules of the target product (surfactin) P are generated per molecule NRPS, as described by Vahidinasab et al. [39]. The terms M_P_ and M_NRPS_ correspond to the molecular weights of the product P and the NRPS enzyme complex, respectively. Finally, *Y*_*NRPS/X*_ describes the production yield of NRPS per unit of biomass formed.

7 $$\:{q}_{P}=\:\alpha\:\:\cdot\:\:\frac{{M}_{P}}{{M}_{NRPS}}\:\cdot\:\:{Y}_{NRPS/X}\:\cdot\:\:\mu\:$$

### Statistical evaluation

For assessing the model`s performance, the RMSE (Root Mean Square Error) was used. Because RMSE has the same unit as the evaluated variables, deviations between experimental measurements and model predictions can be interpreted directly. In an ideal case, the RMSE approaches zero, reflecting an excellent model fit. Moreover, RMSE is sensitive to outliers, allowing pronounced mismatches between predicted and observed values to be detected effectively. This sensitivity enhances the reliability of the model evaluation [40].

### Parameter fitting algorithm

Model parameters were estimated by nonlinear least-squares optimization using MTALAB R2023a. Experimental time-series data for biomass, substrate, and, when applicable, product concentrations were used as reference values for the fitting procedure. A custom objective function calculated the differences between experimental and modelling data at identical time points, with each variable normalized to avoid weighting effects caused by different units or magnitudes. Depending on the strain (BMV9, BMV12, or BMV33), a defined subset of physiological parameters was selected for optimization. The solver “lsqnonlin” was applied with stringent convergence settings and biologically meaningful parameter bounds. When numerical instabilities occurred during modelling, a penalty term ensured that such parameter sets were rejected. After optimization, the modelling was started again using the best-fit parameter values, and the agreement between model predictions and experimental data was quantified using RMSE values for each state variable. This workflow ensured robust and reproducible parameter estimation across all strains.

### Plotting of experimental data and model fits

All graphs were generated with OriginPro 2022b (Origin-Lab Corporation, Northampton, USA) software.

## Results

### Theoretical yield calculation and ATP requirement for *de novo* surfactin synthesis in *B. subtilis*

Yield calculation is crucial for the industrial-scale production of secondary metabolites by bacteria. In Table 2, the redox equivalents for biosynthesis of the fatty acid chain and the amino acids involved in the structure of surfactin were determined and then summed up to estimate the theoretical energy requirement for biosynthesis of one mole surfactin. This analysis focuses exclusively on surfactin biosynthesis and does not include cellular maintenance energy requirements.

In total, for biosynthesis of one mole surfactin, 26 moles NAD(P)H, 10 moles ATP and 12 CO_2_ will be excessed out of the consumption of 12.5 moles of glucose as the substrate for biosynthesis. This results in a theoretical yield of 0.08 moles surfactin/mole glucose or 0.46 g_surfactin_/g_glucose_. Details on the single steps with the respective redox equivalents and the details on theoretical yields calculations are available in supplementary file 1.

**Table 2:** Redox equivalent and needed glucose molecules for the production of 1 molecule surfactin

	Precursor	Redox-equivalent	Required glucose molecules
Formation of 7 amino acids involved in surfactin structure	Glutamate	+ 3 NADPH 0 ATP	1
	Aspartate	+ 2 NADPH 0 ATP	0.5
	Valine	+ 1 NADPH + 2 ATP	1
	4x Leucine	+ 16 NADPH + 12 ATP	6
Formation of fatty acid involved in surfactin structure	3-Hydroxy-13-methytetradecaonic acid	+ 4 NADPH + 3 ATP	4
Activation of seven amino acids		-7 ATP	
Sum		+ 26 NADPH + 10 ATP	

### Growth behavior of strains differing in srfA operon configuration

**Fig. 2:**
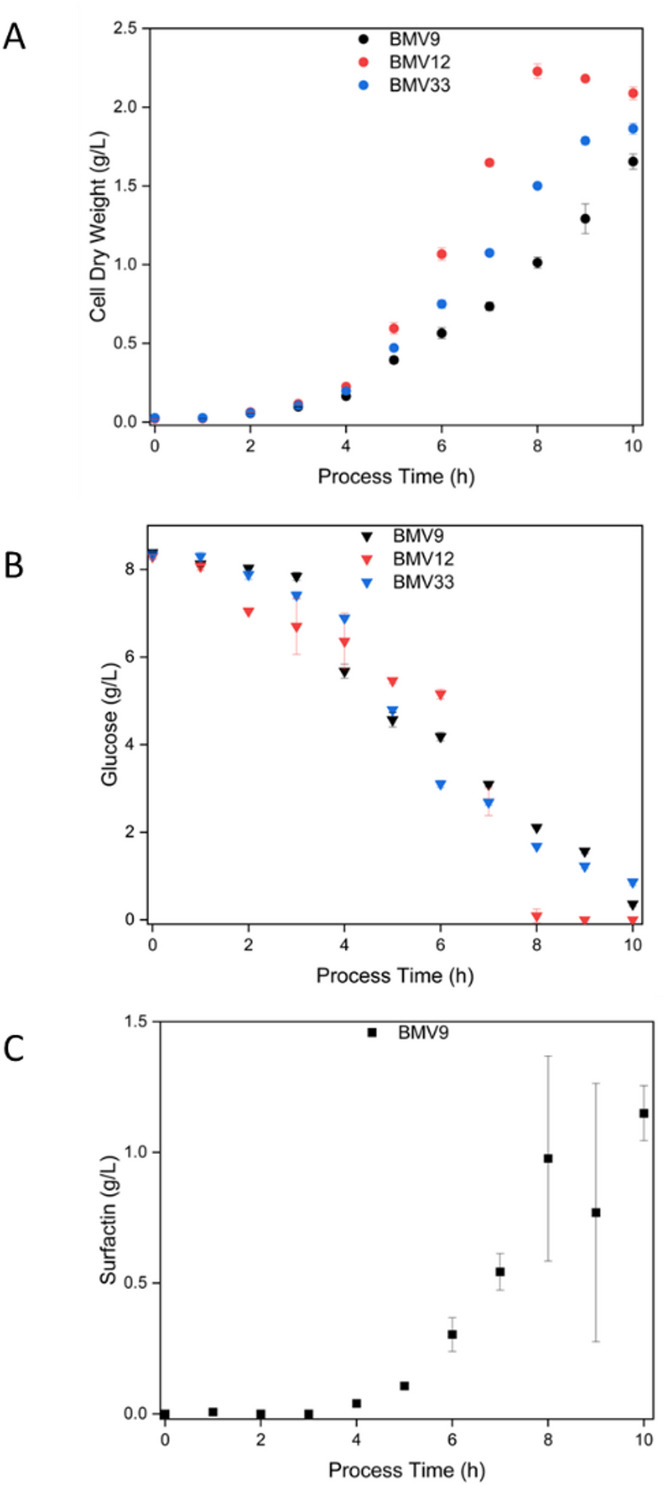
Bacterial growth (cell dry weight, g/L) (**A**), glucose consumption (g/L) (**B**), and surfactin production (g/L) (**C**) of the *B. subtilis* strains BMV9 (surfactin producer – black), BMV12 (Δ*srfAA-AD* – red), and BMV33 (Δ*sfp* – blue) during 10 h of cultivation. Error bars are represented by standard deviation

Due to the very large size of the *srfA* operon (~ 26 kb) encoding the surfactin-forming NRPS in *B. subtilis* a significant metabolic burden is reasonable for the production strain. To get a more detailed overview about the impact associated with the *srfA* operon expression and the subsequent surfactin production in gram-scale, the growth behaviour, glucose and nitrogen consumption of the surfactin-producing *B. subtilis* reference strain BMV9 and two mutant strains, *B. subtilis* BMV12 and BMV33, which do not produce surfactin, were compared (Fig. 2). In BMV12, the entire *srfA* operon is deleted, leading to a reduced metabolic burden due to the non-expression of the comprising operon. In contrast, the surfactin-forming NRPS enzyme is expressed in BMV33, while the *sfp* gene responsible for post-translational activation of the NRPS is deleted, resulting in metabolic burden in *srfA* operon expression but an absence in surfactin synthesis.

Across the cultivation period, BMV12 showed the fastest growth and reached the highest CDW (2.23 g/L), entering stationary phase approximately 8 h after inoculation (hai) (Fig. 2a). Correspondingly, BMV12 exhibited a significantly higher specific growth rate (µ = 0.178 h^− 1^), whereas no significant difference was observed between BMV9 and BMV33 (µ = 0.168 h^− 1^ and 0.170 h^− 1^, respectively). Its growth advantage is consistent with its genetic setup, as the strain does not express the energetically costly *srfA* operon. In contrast, BMV9 and BMV33 displayed slower biomass formation and reached lower final CDW values (1.66 and 1.86 g/L, respectively).

The glucose consumption profiles supported these observations (Fig. 2b). BMV12 initiated substrate uptake earlier and more rapidly; by around 3 hai, its glucose consumption was already higher than that of the other two strains. By 8 hai, BMV12 had consumed more than 97% of the available glucose. At the same time point, both BMV9 and BMV33 remained below 80% glucose consumption, reflecting their slower growth rates. BMV9 and BMV33 continued utilizing glucose gradually throughout the later stages of cultivation.

Surfactin production was only detected for the reference strain BMV9 (Fig. 2c). Surfactin accumulation became evident after 4 hai and increased steadily toward the end of cultivation. No surfactin was detected for BMV12 or BMV33, as expected based on their genetic deletions.

### Proteome analysis

To get more insight into the molecular adaptation processes in the constructed mutant strains compared to the reference strain *B. subtilis* BMV9, proteome analyses were performed. Samples were taken from the late exponential phase, allowing cell differentiation processes in all the *B. subtilis* strains.

**Fig. 3:**
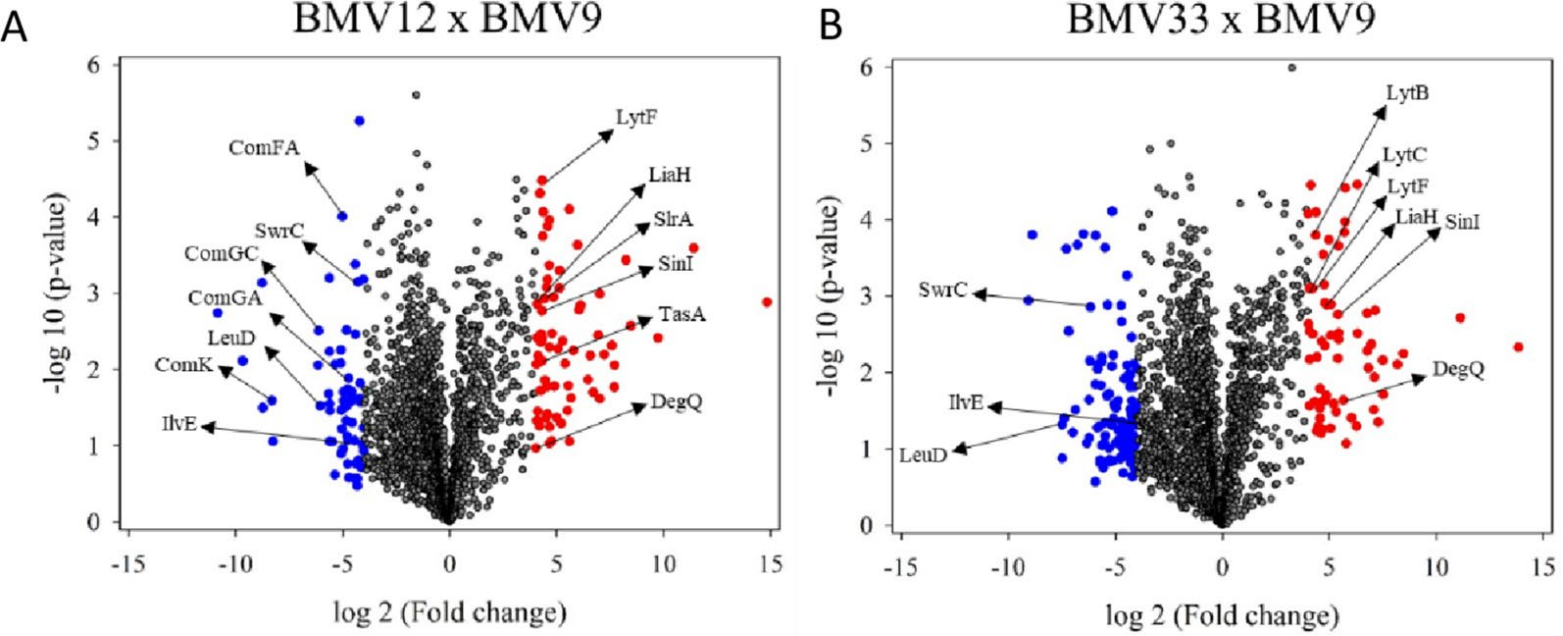
Differential proteome analysis of *Bacillus subtilis* mutant strains lacking surfactin production

Specifically, the mutant strains BMV12 (Δ*srfAA-AD*) and BMV33 (Δ*sfp*) displayed overall comparable proteome profiles, reflecting their shared inability to synthesize surfactin. Nevertheless, distinct differences related to their genetic configurations were observed. In BMV12, a clear reduction in competence-associated proteins was detected compared to BMV33. In particular, the abundance of the competence master regulator ComK and key components of the DNA uptake machinery, including the pseudopilus ATPase ComGA, the major pseudopilin ComGC and the DNA translocation ATPase ComFA, were significantly reduced. This observation is consistent with the deletion of the *comS* gene embedded within the *srfAB* locus in BMV12, which normally stabilizes ComK and promotes activation of the competence regulon (Fig. 3).

Volcano plots showing significantly regulated proteins in BMV12 (Δ*srfAA-AD*) (A) and BMV33 (Δ*sfp*) (B) compared to the surfactin-producing reference strain BMV9. The x-axis represents log_2_ fold change in protein abundance, while the y-axis indicates statistical significance as -log_10_(p-value). Proteins with significant downregulation are highlighted in blue and upregulated proteins in red, whereas non-significant proteins are shown in grey. Selected proteins involved in competence (ComK, ComGA, ComGC, ComFA), precursor metabolism (IlvE, LeuD), surfactin resistance (SwrC), secretion (LiaH), biofilm regulation (SinI, SlrA), and cell envelope remodelling (Lyt proteins) are indicated, more details can be found in the supplementary table S1.

In contrast, both mutant strains showed several common proteomic responses associated with the absence of surfactin production. Proteins involved in the biosynthesis of branched-chain amino acids, including Ilv- and Leu-proteins required for leucine formation, were detected at reduced abundance in both BMV12 and BMV33 (Fig. 3). Because leucine represents a major building block of the surfactin peptide structure, this decrease likely reflects a rebalancing of precursor pathways when surfactin biosynthesis is inactive. Consistently, the surfactin self-resistance protein SwrC was also reduced in both strains compared to the surfactin-producing reference strain BMV9.

Conversely, several proteins associated with secretion and cell envelope processes were more abundant in the non-surfactin-producing mutants. In particular, the twin-arginine translocation pathway component LiaH showed increased abundance, suggesting enhanced protein and peptide export capacity. Similarly, Lyt-proteins involved in cell envelope remodelling, cell division, and cell wall turnover were detected at higher levels in both mutant strains.

Finally, regulatory proteins associated with biofilm formation were affected. The SinR antagonists SinI and SlrA (the latter specifically in BMV12) were detected with increased abundance, which is consistent with the elevated levels of TasA, a major component of the biofilm matrix. Together, these findings suggest that the absence of surfactin production may favor cellular differentiation processes associated with biofilm formation.

Additional deregulated proteins were related to motility and developmental pathways, including several flagellar proteins (FlgM, FliG, FliH, FliJ, FliP and FlhA) as well as sporulation-associated proteins (KinD, KinE, SafA, SpoIISB, SspE and SspP). Moreover, the phenolic acid decarboxylase BsdD, which participates in alternative amino-acid metabolism and redox-related pathways, was specifically increased in BMV33. The regulatory protein DegQ, known to influence extracellular protease activity and lipopeptide production, was also detected with increased abundance. A complete overview of the proteomics dataset is provided in supplementary table S1.

### Modelling results

#### Model parametrization

The parametrization of the kinetic model provided strain-specific parameter sets that describe the cultivation behavior of BMV12, BMV33, and BMV9 (Tables 3, 4 and 5). The fitted maximum specific growth rate (µ_max_) decreased from 0.623 h^-1^ for BMV12 to 0.553 h^-1^ BMV33 and further to 0.501 h^-1^ BMV9. In addition, the estimated yield for substrate-to-biomass (Y_X/S_) conversion decrease from 0.269 g/g for BMV12 to 0.249 g/g for BMV33 and 0.206 g/g for BMV9, indicating strain-dependent differences in substrate utilization.

**Table 3:** Parameters with ranges for the modelling of the shake flask experiments for the *Bacillus subtilis* strain BMV12

Parameter	Value	Unit	Range	Comment/source
µ_max_	0.623	1/h	0.05-2	Fitting parameter
K_S_	0.05	g/L	–	Hiller et al. [16]
m	0.05	g/(g*h)	–	Hiller et al. [16]
t_lag_	0.0335	h	0–5	Fitting parameter
Y_X/S_	0.269	g/g	–	Experimental value
i	12	–	–	Strain

**Table 4:** Parameters with ranges for the modelling of the shake flask experiments for the *Bacillus subtilis* strain BMV33

Parameter	Value	Unit	Range	Comment/source
µ_max_	0.553	1/h	0.05-2	Fitting parameter
K_S_	0.05	g/L	–	Hiller et al. [16]
m	0.05	g/(g*h)	–	Hiller et al. [16]
t_lag_	0.06101	h	0–5	Fitting parameter
Y_X/S_	0.249	g/g	–	Experimental value
Y_NRPS/X_	0.0699	g/g	0-0.1	Fitting parameter
Y_NRPS/S_	0.0696	g/g	0–1	Fitting parameter
i	33	–	–	Strain

**Table 5:** Parameters with ranges for the modelling of the shake flask experiments for the *Bacillus subtilis* strain BMV9

Parameter	Value	Unit	Range	Comment/source
µ_max_	0.501	1/h	0.05-2	Fitting parameter
K_S_	0.05	g/L	–	Hiller et al. [16]
m	0.05	g/(g*h)	–	Hiller et al. [16]
t_lag_	0.117	h	0–5	Fitting parameter
Y_X/S_	0.206	g/g	–	Experimental value
Y_P/S_	0.46	g/g	–	Stoichiometry
Y_NRPS/X_	0.0699	g/g	–	Fixed from BMV33
Y_NRPS/S_	0.0696	g/g	–	Fixed from BMV33
α	11,285	Molecules/molecule	1270–14,348	Fitting parameter; Vahidinasab et al. [39]
M_P_	1036.34	g/mol	–	Calculated
M_NRPS_	974,583	g/mol	–	Calculated
i	9	–	–	Strain

#### Modelling of shake flask experiments

**Fig. 4:**
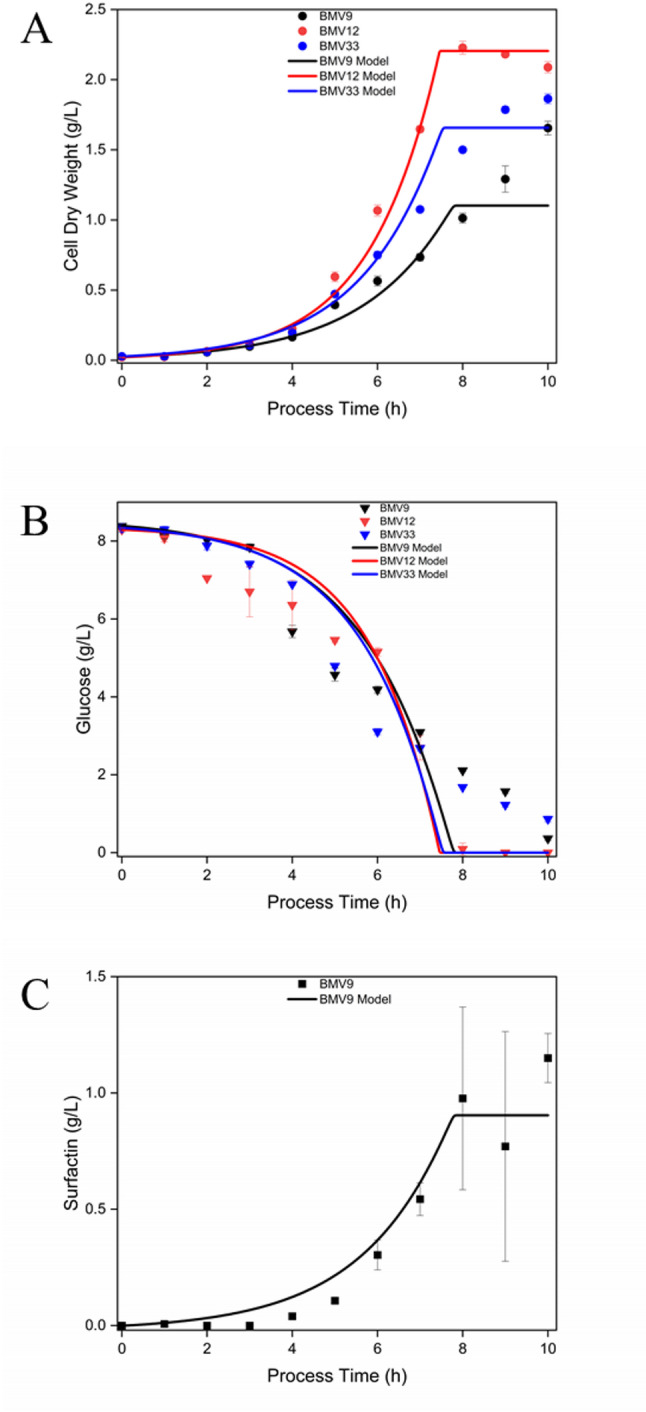
Experimental data (symbols) and model fits (lines) of shake flask cultivations of the *Bacillus subtilis*, strains BMV9 (surfactin producer – black), BMV12 (Δ*srfAA-AD* – red), and BMV33 (Δ*sfp* – blue) during 10 h. Bacterial growth (cell dry weight, g/L) (**A**), glucose consumption (g/L) (**B**), and surfactin production (g/L) (**C**) over process time. Error bars are represented by standard deviation

The kinetic model was applied to describe biomass formation, substrate consumption, and surfactin production during shake flask cultivations of the investigated strains, based on the experimental dataset provided in supplementary table S2. The modelling showed good agreement with the experimental data for all state variables (Fig. 4). In particular, the model captured the exponential growth phase, the subsequent transition into stationary phase, and the overall trends in glucose consumption across all strains. Surfactin formation in BMV9 was also adequately represented, including the onset and progression of product accumulation.

A slight deviation between experimental and simulated glucose concentrations was observed during the stationary phase, particularly for BMV9 and BMV33. This discrepancy may indicate the presence of non-growth-associated glucose consumption that is not fully represented by the current model structure. Possible contributors include maintenance energy requirements, protein turnover, stress-response mechanisms, and ongoing secondary metabolic activities. Consequently, glucose consumption during stationary phase may not be exclusively linked to biomass formation and product synthesis, which likely contributes to the observed deviation between model predictions and experimental data.

The kinetic model adequately described biomass growth, glucose consumption, and surfactin formation for all investigated strains (Fig. 4), it was further supported by the calculated RMSE values (Table 6).

**Table 6:** Root mean squared error (RMSE) values of the kinetic model for the process parameters biomass X, substrate (glucose) S, and product (surfactin) P for the *Bacillus subtilis* strains BMV9, BMV12, and BMV33

Strain	RMSE X	RMSE S	RMSE *P*
BMV9	0.184	1.11	0.1031
BMV12	0.0749	0.686	–
BMV33	0.1061	0.993	–

### Quantification of metabolic burden associated with NRPS expression and surfactin production

To further quantify the physiological impact of surfactin biosynthesis, the observed differences in maximum specific growth rates were used to estimate the relative metabolic burden associated with NRPS expression and surfactin production. The strain BMV12 (Δ*srfAA-AD*) was considered to represent the baseline growth capacity (µ_max,0_) of the system. In contrast, strain BMV33 expresses the large NRPS complex but does not produce surfactin. The reduction in growth rate between BMV12 and BMV33 therefore reflects the metabolic cost associated with expression, translation, and maintenance of the NRPS machinery itself. Based on the measured growth rates, this difference corresponds to a decrease of Δµ_NRPS_ = 0.07 h^− 1^ (µ_max,0_ – µ_max, BMV33_), which represents 11.23% of the baseline growth capacity.

A further reduction in growth rate was observed for the surfactin-producing strain BMV9, which carries both the functional *srfA* operon and the *sfp* gene, enabling active surfactin biosynthesis. The difference between BMV33 and BMV9 therefore reflects the additional metabolic burden associated with the actual production of surfactin (µ_prod_), including precursor diversion and energy demand for biosynthesis. This additional penalty was estimated as Δµ_prod_ = 0.052 h^− 1^, corresponding to 8.34% of the baseline growth rate. Taken together, these results indicate that the overall metabolic burden associated with surfactin production under the tested conditions represents roughly 19.57% of the potential growth capacity of the cell, with the expression of the large NRPS machinery accounting for the majority of the observed growth penalty.

## Discussion

The results of this study demonstrate that surfactin biosynthesis imposes a measurable physiological burden on *B. subtilis*, affecting growth behavior, metabolic allocation, and proteome composition. By comparing the surfactin-producing strain BMV9 with two non-producing mutants differing in their genetic configuration (*ΔsrfAA-AD* and *Δsfp*), it was possible to dissect the relative contributions of NRPS expression and active surfactin synthesis to the overall metabolic burden. Such an approach provides a clearer understanding of the physiological costs associated with the production of complex secondary metabolites in bacteria.

### NRPS expression and surfactin biosynthesis

Surfactin is synthesized by a large NRPS complex encoded by the *srfA-AD* operon. NRPS systems operate as modular assembly lines in which each module catalyzes the activation and incorporation of specific amino acid residues into the growing peptide chain. This modular organization allows the biosynthesis of structurally diverse secondary metabolites and represents one of the most complex enzymatic systems found in bacteria [41, 42].

The surfactin synthetase complex of *B. subtilis* consists of multiple megadalton-sized subunits that sequentially catalyze peptide elongation and cyclization reactions. Due to their large size and complex catalytic architecture, the synthesis and maintenance of NRPS enzymes impose a considerable metabolic burden on the producing cells. Consequently, the regulation of NRPS expression is a key factor influencing surfactin productivity.

Recent quantitative proteomic analyses have shown that the abundance of surfactin-forming NRPS complexes can vary considerably during cultivation and may represent a limiting factor for biosurfactant productivity [39]. These observations further support the idea that both the expression level and catalytic activity of the NRPS machinery must be considered when evaluating the physiological costs of surfactin biosynthesis.

The comparison of the three isogenic strains demonstrated that expression of the *srfA* operon alone already reduces cellular growth capacity. The strain BMV33, which expresses the NRPS machinery but lacks the *sfp* gene required for post-translational activation, showed a lower specific growth rate than the strain BMV12 lacking the entire *srfA* operon. This observation indicates that the transcription, translation, and maintenance of the large NRPS complex impose a measurable metabolic cost even in the absence of surfactin formation. Considering the large size of the *srfA* operon (~ 26 kb) and the total protein mass of the NRPS complex exceeding 900 kDa, such a resource investment is consistent with the high biosynthetic demands of modular peptide synthetases described previously [41, 42].

An additional reduction in growth was observed for the surfactin-producing strain BMV9, demonstrating that the biosynthesis of the lipopeptide itself imposes further metabolic constraints. Based on the growth rate differences between the three strains, the expression of the NRPS machinery accounted for approximately 11% of the total growth penalty, while the active biosynthesis of surfactin contributed an additional reduction of approximately 8% in growth capacity. Together, these findings indicate that nearly one fifth of the potential cellular growth capacity may be redirected toward the expression of the surfactin biosynthetic machinery and the formation of the lipopeptide under the tested conditions.

These physiological observations are supported by the theoretical calculations of precursors and energy requirements performed in this study (Table 2). The biosynthesis of one mole of surfactin requires significant amounts of reducing power and ATP, including 26 mol NADPH and 10 mol ATP for the formation of the peptide and fatty acid precursors.

In addition, seven ATP molecules are required for the activation of amino acids by the NRPS modules during peptide assembly. The high energetic demand and the diversion of central metabolic precursors therefore provide a mechanistic explanation for the reduced growth rates observed in the surfactin-producing strain. Although the present study cannot exclude minor contributions from pathway-specific biosynthetic intermediates, NRPS systems generally retain intermediates covalently bound to carrier domains during peptide assembly [42], suggesting that the observed burden primarily originates from the energetic and metabolic costs of enzyme expression and product formation rather than from intermediate toxicity.

Nevertheless, the growth differences observed for BMV33 should be interpreted with caution, as deletion of *sfp* may affect additional Sfp-dependent secondary metabolic pathways beyond surfactin biosynthesis.

### Regulation of surfactin production

The expression of the *srfA* operon is tightly regulated by quorum sensing and global transcriptional regulators that coordinate the transition from primary to secondary metabolism. In *B. subtilis*, the ComQXPA quorum-sensing system activates the competence transcription factor ComK, which plays an important role in the regulation of competence development and indirectly influences surfactin production [43, 44]. In addition, the *comS* gene, which is embedded within the *srfAB* locus, stabilizes ComK and thereby promotes activation of the competence regulon [9].

The proteomic results obtained in this study support this regulatory connection. In the strain BMV12, which also lacks the *comS* gene located within the *srfAB* region, a significant reduction in competence-related proteins was observed (ComK, ComGA, ComGC and ComFA) compared with BMV33 and BMV9 (Fig. 3). This observation is consistent with the known role of ComS in stabilizing ComK and maintaining competence gene expression.

Additional regulatory elements involved in surfactin production have also been described. For example, the protein SwrC is associated with surfactin secretion and self-resistance, protecting producing cells from the detergent-like activity of the lipopeptide [45]. In agreement with the absence of surfactin biosynthesis in BMV12 and BMV33, the abundance of SwrC was reduced in both strains, indicating that surfactin resistance mechanisms are downregulated when the lipopeptide is not produced.

Beyond transcriptional regulation, stress-response pathways can also influence surfactin-related cellular processes. The LiaRS two-component system controls gene expression associated with cell envelope stress responses in *B. subtilis* [46]. Consistently, the proteomic analysis revealed increased abundance of the LiaH protein in both non-surfactin-producing strains, suggesting enhanced activation of the LiaRS-dependent stress response and potentially altered protein export or membrane-associated processes when surfactin biosynthesis is inactive.

The interpretation of these changes should consider the genetic background of the investigated strains. All strains used in this study are derived from the surfactin production strain JABs32 (3NA-derived) and therefore carry the characteristic *spo0A* mutation and elongated *AbrB* regulator, resulting in a strongly reduced capacity for sporulation and cellular differentiation [11, 12, 22]. Consequently, the observed proteomic differences are unlikely to be explained by major shifts in differentiation-associated subpopulations, as would be expected in wild-type *B. subtilis* populations.

Instead, the increased abundance of LiaH and several Lyt proteins may indicate alterations in cell-envelope homeostasis associated with the absence of surfactin production. Surfactin is a membrane-active lipopeptide that interacts with biological membranes and can influence membrane properties in a concentration-dependent manner [47]. Consistent with this interpretation, both non-surfactin-producing strains exhibited reduced abundance of the surfactin self-resistance protein SwrC, indicating a reduced requirement for surfactin-associated membrane protection mechanisms. While the present data do not allow us to conclude whether surfactin production itself directly induces cell-envelope stress, the observed proteomic changes are consistent with alterations in envelope homeostasis associated with the presence or absence of surfactin biosynthesis.

### Surfactin and multicellular behavior

Surfactin production plays a central role in the multicellular behavior of *B. subtilis*. In addition to its well-known biosurfactant properties, surfactin also functions as a signaling molecule that influences biofilm formation and cellular differentiation [48, 49].

It has been demonstrated that surfactin can trigger potassium leakage from the cell membrane, which subsequently activates the histidine kinase KinC and initiates signaling pathways involved in multicellular community development [50]. This signaling cascade contributes to the formation of complex biofilm structures in *B. subtilis*, where genetically identical cells differentiate into distinct subpopulations performing specialized physiological roles [51]. In particular, surfactin-producing cells can induce matrix-producing cells within the population, thereby coordinating the development of structured bacterial communities.

Consistent with the regulatory link between surfactin production and multicellular behavior, the proteomic analysis performed in this study revealed increased abundance of proteins associated with biofilm regulation in the non-surfactin-producing mutant strains (Fig. 3). In particular, the SinR antagonists SinI and SlrA were detected at higher levels, with SlrA specifically increased in BMV12. These proteins relieve the repression imposed by the master biofilm regulator SinR, thereby promoting the expression of biofilm matrix genes. Accordingly, the biofilm matrix protein TasA was also detected at increased abundance in BMV12.

Additional evidence for the close connection between surfactin biosynthesis and cellular developmental pathways was provided by the altered abundance of several flagellar and sporulation-associated proteins. The observed changes did not indicate a uniform repression of these processes but rather a complex pattern of up- and downregulated proteins. This suggests that the effects are primarily regulatory rather than a direct consequence of precursor limitation. Surfactin is known to play an important role in swarming motility and multicellular behavior, and alterations in surfactin biosynthesis can therefore influence the expression of motility-associated proteins [52, 53]. Since all investigated strains are derived from the sporulation-deficient strain JABs32, the observed changes in sporulation-related proteins are more likely to reflect regulatory adjustments within differentiation-associated networks than active sporulation processes.

These observations suggest that the absence of surfactin production may alter the regulatory balance controlling cellular differentiation processes in *B. subtilis*. While surfactin normally acts as a signaling molecule coordinating multicellular behavior, the lack of surfactin synthesis in BMV12 and BMV33 appears to be accompanied by compensatory changes in regulatory pathways associated with biofilm formation and community development.

### Metabolic constraints in surfactin biosynthesis

Several studies have investigated metabolic bottlenecks affecting surfactin production. Because surfactin contains both peptide and fatty acid moieties, its synthesis requires a coordinated supply of amino acids and lipid precursors derived from central carbon metabolism. In particular, branched-chain amino acids play a crucial role, as leucine occurs four times within the cyclic heptapeptide structure of surfactin. Metabolic modelling approaches have therefore identified leucine biosynthesis as an important control point influencing surfactin formation [19].

The proteomic results obtained in this study support the central role of branched-chain amino acid metabolism. Both non-surfactin-producing strains (BMV12 and BMV33) exhibited reduced abundance of proteins involved in leucine biosynthesis, including Ilv- and Leu-associated enzymes (Fig. 3). This reduction likely reflects a decreased cellular demand for branched-chain amino acids when surfactin biosynthesis is inactive. The observed downregulation therefore suggests a metabolic rebalancing of precursor pathways when the surfactin synthesis pathway is not operating.

In addition to precursor availability, surfactin biosynthesis also requires substantial energetic investment. The theoretical calculations performed in this study indicate that the biosynthesis of one mole of surfactin requires 26 mol NADPH and 10 mol ATP for the formation of the amino acid and fatty acid precursors (Table 2). Furthermore, the NRPS-mediated peptide assembly requires additional ATP molecules for the activation of each amino acid residue. This high demand for reducing power and energy highlights the considerable metabolic investment required for lipopeptide biosynthesis.

Together with the growth-rate-based burden analysis, these calculations suggest that the physiological costs of surfactin production originate primarily from three factors: (i) the energetic demand for precursor synthesis, including ATP and NADPH consumption, (ii) the diversion of amino acid and fatty acid precursors away from biomass formation, and (iii) the expression, translation, and maintenance of the large NRPS machinery. While potential membrane-associated effects of surfactin cannot be excluded, the present data indicate that resource allocation and biosynthetic investment represent the dominant contributors to the observed metabolic burden.

Recent metabolic engineering approaches have therefore attempted to redirect cellular resources toward surfactin production. For example, targeted repression of amino acid biosynthesis pathways using CRISPR interference has been shown to enhance surfactin titers by reallocating metabolic flux toward lipopeptide biosynthesis [20]. However, precursor supply alone may not fully explain the limitations of surfactin production. Recent studies have further suggested that the abundance of active NRPS complexes may itself represent a critical limiting factor in lipopeptide biosynthesis [39].

The abundance and expression of the NRPS machinery itself can represent an additional metabolic constraint. Even in the absence of surfactin synthesis, expression of the large NRPS complex in strain BMV33 was associated with a measurable growth penalty compared with the operon-deleted strain BMV12. This observation suggests that the transcription and translation of the large biosynthetic gene cluster already impose a substantial metabolic burden, independent of the catalytic activity of the enzyme complex. Consequently, both precursor supply and biosynthetic machinery expression must be considered when evaluating metabolic limitations in surfactin production.

### Interaction between lipopeptide pathways

It should be noted that the deletion of *sfp* in BMV33 does not exclusively abolish surfactin production. As Sfp is a broad-spectrum 4′-phosphopantetheinyl transferase required for the post-translational activation of carrier proteins involved in secondary metabolism, its absence may also impair the biosynthesis of additional lipopeptides and other carrier-protein-dependent metabolites [54]. Therefore, the physiological differences observed between BMV33 and the other strains cannot be attributed solely to the lack of surfactin formation, but may also reflect the loss of other Sfp-dependent metabolic functions, such as others NRPS (e.g. fengycin and bacillibactin), apo-acyl carrier proteins (ACPs) (e.g. AcpP) of polyketide synthases (PKSs) (e.g. Bacillaene and difficidin), and fatty acid synthases (FASs) (e.g. ACP-dependent type II FASs) [8, 12, 39, 55–58]. Increasing evidence suggests that these pathways are interconnected at both the metabolic and regulatory levels. For instance [12], demonstrated that deletion of the surfactin synthetase operon can significantly alter plipastatin production, indicating that these lipopeptide biosynthetic systems are not independent but rather share regulatory networks and metabolic resources.

Such interactions likely arise from the shared use of metabolic precursors, including branched-chain amino acids and fatty acid intermediates, as well as overlapping regulatory networks controlling secondary metabolism. The regulation of lipopeptide biosynthesis in *B. subtilis* is coordinated by global transcriptional regulators and quorum-sensing pathways that integrate environmental signals with cellular metabolic status.

Consistent with this concept, the proteomic analysis revealed alterations in regulatory proteins associated with lipopeptide production. In particular, the regulatory protein DegQ showed increased abundance in the mutant strains (Fig. 3). DegQ is known to influence extracellular protease production and has previously been associated with changes in lipopeptide biosynthesis in *B. subtilis* [59]. The observed increase in DegQ abundance may therefore reflect a broader regulatory adjustment of secondary metabolism when surfactin production is absent.

### Implications for process optimization

Understanding the regulation and physiological consequences of surfactin biosynthesis is essential for improving industrial production processes. The results presented here demonstrate that both the expression of the large NRPS machinery and the biosynthesis of surfactin itself impose a substantial metabolic burden on *B. subtilis*, redirecting a considerable fraction of the cellular growth capacity toward secondary metabolite production. Consequently, strategies aimed at improving surfactin yields must balance production efficiency with the physiological limitations of the host organism.

The burden analysis presented here also provides guidance for future strain engineering efforts. Since a substantial fraction of the observed burden originates from expression and maintenance of the large NRPS machinery, strategies enabling dynamic or growth-phase-dependent control of *srfA* operon expression may improve overall process performance. Such approaches would allow biomass accumulation during the initial growth phase while postponing the metabolic costs associated with surfactin biosynthesis to later stages of cultivation.

In addition, the observed alterations in amino acid metabolism suggest that further optimization of precursor supply pathways, particularly those involved in branched-chain amino acid biosynthesis, may contribute to improved surfactin yields. More generally, quantitative assessment of metabolic burden provides a useful framework for evaluating trade-offs between cellular fitness and product formation during microbial cell factory development.

The findings suggest that process optimization strategies should focus not only on increasing precursor supply but also on controlling the timing and intensity of *srfA* operon expression. For example, decoupling biomass formation from surfactin production, through inducible expression systems or two-stage cultivation strategies, may allow cells to first accumulate biomass before redirecting metabolic resources toward lipopeptide biosynthesis.

Future engineering studies should also address the contribution of other Sfp-dependent metabolites to the observed phenotype. Targeted deletion of additional Sfp-dependent biosynthetic pathways, such as plipastatin/fengycin, bacillibactin, or polyketide-related pathways, would allow a more precise separation of the metabolic burden caused specifically by surfactin biosynthesis from the broader burden associated with Sfp-dependent secondary metabolism.

Another promising strategy is the use of genome-reduced strains. By removingdispensable genomic regions, genome reduction may decrease unnecessary cellular resource allocation and reduce interference from competing metabolix pathways. Recent work showed that a genome-reduced *B. subtilis* Δ6 strain produced higher surfactin levels than its parental strain, despite lower biomass formation, suggesting a redirection of cellular resources toward product synthesis [60]. Therefore, genome streamlining could be further explored as a complementary approach to dynamic pathway regulation and precursor-supply optimization.

In addition, the observed proteomic adaptations highlight that surfactin production is embedded within a broader regulatory network influencing amino acid metabolism, stress responses, and cellular differentiation processes. These interactions emphasize the importance of systems-level approaches in strain engineering and bioprocess optimization, where both metabolic pathway engineering and regulatory network modulation must be considered to achieve efficient and stable surfactin production.

## Supplementary Information

Supplementary Material 1.

Supplementary Material 2.

Supplementary Material 3.

## Data Availability

Data Availability Statement: The datasets generated and analyzed during the current study are stored on secure university servers and are not publicly available due to institutional data man-agement and storage policies. Access to the data may be granted by the corresponding author upon reasonable request.
