# Supplementary material for: Exploring the metabolic burden of surfactin biosynthesis and the metabolic costs of srfA operon expression in Bacillus subtilis
Source: Microb Cell Fact. 2026 Jun 19;25:145. doi: 10.1186/s12934-026-03047-1 (PMC13289377; doi:10.1186/s12934-026-03047-1)
Supplement: Supplementary file 2 — Supplementary Material 2. [file 12934_2026_3047_MOESM2_ESM.docx]

**Supplementary file 1**

**Exploring the metabolic burden of surfactin biosynthesis and the metabolic costs of *srfA* operon expression in *Bacillus subtilis***

Elvio Henrique Benatto Perino^1+*^, Eric Hiller^1,+,*^, Maliheh Vahidinasab^1^, Sandra Moneta^1^, Bahar Abrishamchi^1^, Kowsala Nagendran^1^, Philipp Hubel^2^, Jens Pfannstiel^2^, Rudolf Hausmann^1^

^1^Department of Bioprocess Engineering (150k), Institute of Food Science and Biotechnology, University of Hohenheim, Fruwirthstrasse 12, 70599 Stuttgart, Germany

^2^Core Facility Hohenheim, Mass Spectrometry Unit, University of Hohenheim, August-von-Hartmann-Str. 3,

70599 Stuttgart, Germany

^+^Contributed equally

*Corresponding authors

Email: eperino@uni-hohenheim.de and eric.hiller@uni-hohenheim.de

Telephone: +49 711 45924726

**Table S1. List of the primers used in this study**

| **Primer** | **Sequence 5’→ 3’** | **Application** |
| --- | --- | --- |
| *sfp*_01 Forward | GTT ATC CTT GCT TTG TGC CAT GC | Amplification of *sfp*:*loxP-ermC*:*loxP* region in BKE03570 with upstream and downstream flanking sites |
| *sfp*_02 Reverse | CGC CCT TCT GTC AGA TGT GC |  |
| *sfp*_03_conf Forward | GAA AGC TCT TGG CCA CTC AAC AG | Confirmation of transformation |
| *sfp*_04_conf Reverse | GGT TTG CCA ACA CGC TGA AAC |  |

Calculating yield is essential for the industrial production of secondary metabolites, such as surfactin, by bacteria. In determining the energy balance for surfactin biosynthesis in Bacillus subtilis, the primary glucose metabolism pathways glycolysis and the TCA cycle were analyzed. This analysis also included the subsequent precursor biosynthesis and the energy demands for amino acid activation and peptide bond formation via the NRPS enzyme complex. The following sections outline the redox equivalents involved in glycolysis and the TCA cycle, as well as the amino acids and fatty acids that contribute to the structure of surfactin.

During glycolysis, 2 moles of ATP, 2 moles of NAD(P)H, and 2 moles of pyruvate are generated. One of the pyruvate molecules is carboxylated to produce oxaloacetate, a process that consumes one mole of ATP. The second pyruvate is converted into acetyl-CoA. The resulting oxaloacetate and acetyl-CoA then combine to form citrate. Typically, one mole of pyruvate can be fully oxidized in the TCA cycle, producing 4 moles of NADH, 1 mole of FADH₂, and 1 mole of GTP.

**Table S2. Steps of glycolysis and TCA with respective redox equivalents**

| **Reaction** | | **Redox equivalent** | **Carbon** |
| --- | --- | --- | --- |
| Glucose | Glucose-6-P | -1 ATP |  |
| Glucose-6-P | Fructose-6-P |  |  |
| Fructose-6-P | Fructose-1,6-BP | -1 ATP |  |
| Fructose-1,6-BP | DHAP |  |  |
| Fructose-1,6-BP | 2x Glyceraldehyde-3-P |  |  |
| 2x Glyceraldehyde-3-P | 2x 1,3-BP-glycrate | +2 NADH |  |
| 2x 1,3-BP-glycrate | 2x 3-P-glycerate | +2 ATP |  |
| 2x 3-P-glycerate | 2x 2-P-glycerate |  |  |
| 2x 2-P-glycerate | 2x PEP |  |  |
| 2x PEP | 2x Pyruvate | +2 ATP |  |
| Pyruvate | Acetyl-CoA | +1 NADH | +1 CO_2_ |
| Acetyl-CoA | Citrate |  |  |
| Citrate | Cis-aconitate |  |  |
| Cis-aconitate | Isocitrate |  |  |
| Isocitrate | 2-Oxogluterate | +1 NADPH | +1 CO_2_ |
| 2-Oxogluterate | Succinyl-CoA | +1 NADH | +1 CO_2_ |
| Succinyl-CoA | Succinate | +1 GTP |  |
| Succinate | Fumarate | +1 FADH |  |
| Fumarate | Malate |  |  |
| Malate | Oxalacetate | +1 NADH |  |

l-Glutamate

The direct precursor for the biosynthesis of l-glutamate, one of the amino acids involved in the structure of surfactin, is 2-oxoglutarate, an intermediate in the TCA cycle. Table S3 outlines the sequence of reactions, beginning with pyruvate and culminating in the conversion of 2-oxoglutarate to l-glutamate. During glycolysis, 2 moles of ATP and 2 moles of NAD(P)H are produced, leading to an overall energy balance of 3 moles of NAD(P)H and no ATP for the conversion of glucose to l-glutamate. Glutamate is crucial for synthesizing the remaining amino acids necessary for surfactin production, with its transamination to 2-oxoglutarate being a key step in the final conversion to these amino acids.

**Table S3. Redox equivalents during the conversion of pyruvate to L-glutamate.**

| **Reaction** | | **Redox equivalent** | **Carbon** |
| --- | --- | --- | --- |
| Pyruvate | Acetyl-CoA | +1 NADH | +1 CO_2_ |
| Pyruvate | Oxalacetate | -1 ATP | -1 CO_2_ |
| Acetyl-CoA  +  Oxalacetate | Citrate |  |  |
| Citrate | Isocitrate |  |  |
| Isocitrate | 2-Oxogluterate | +1 NADPH | +1 CO_2_ |
| 2-Oxogluterate  +  Glutamine | 2x L-Glutamate | -1 ATP  -1 NADPH |  |

L-Aspartate

Oxaloacetate, the direct precursor to aspartate, is an intermediate in the TCA cycle. In this process, 2 moles of pyruvate produced from glycolysis can be carboxylated to form 2 moles of oxaloacetate. Considering the redox equivalents generated during glycolysis (2 moles of ATP and 2 moles of NAD(P)H), the overall metabolic balance for producing 2 moles of l-aspartate is 2 moles of NAD(P)H and 0 moles of ATP. Since only one mole of l-aspartate is needed for surfactin synthesis, this balance can be halved (Table S4).

**Table S4. Redox equivalents during the conversion of pyruvate to L-aspartate.**

| **Reaction** | | **Redox equivalent** | **Carbon** |
| --- | --- | --- | --- |
| 2x Pyruvate | 2x Oxalacetate | -2 ATP | -2 CO_2_ |
| 2x Oxalacetate  +  2x L-Glutamate | 2x L-Aspartate  +  2x 2-Oxogluerate |  |  |

Branched amino acids L-valine and L-leucine

For the branched amino acids l-valine and l-leucine, glucose is metabolized through glycolysis to produce two moles of pyruvate. These 2 moles of pyruvate are then utilized in the synthesis of these amino acids. Table S5 outlines the detailed reaction steps, starting with pyruvate, and includes the necessary or generated redox equivalents for l-valine synthesis.

**Table S5. Redox equivalents during the conversion of pyruvate to L-valine.**

| **Reaction** | | **Redox equivalent** | **Carbon** |
| --- | --- | --- | --- |
| 2-Acetolactate | 2,3-Dihydroxy-3-methylbutanoate | -1 NADPH |  |
| 2,3-Dihydroxy-3-methylbutanoate | 3-Methyl-2-oxobutanoate |  |  |
| 3-Methyl-2-oxobutanoate  +  L-Glutamate | L-Valine  +  2-Oxogluertae |  |  |

Considering the redox equivalents generated during glycolysis (2 moles of NAD(P)H and 2 moles of ATP), the final balance for l-valine synthesis was determined to be 1 mole of NAD(P)H and 2 moles of ATP. For l-leucine, the other branched amino acid involved in surfactin synthesis, 3-methyl-2-oxobutanoate is further metabolized. Table S6 provides details on the redox equivalents produced and consumed during L-leucine synthesis.

**Table S6. Redox equivalents during the conversion of pyruvate to L-leucine.**

| **Reaction** | | **Redox equivalent** | **Carbon** |
| --- | --- | --- | --- |
| 2x Pyruvate | 2-Acetolactate |  | + 1CO_2_ |
| 2-Acetolactate | 2,3-Dihydroxy-3-methylbutanoate | -1 NADPH |  |
| 2,3-Dihydroxy-3-methylbutanoate | 3-Methyl-2-oxobutanoate | -1 Acetyl-CoA | |
| 3-Methyl-2-oxobutanoate | 2-Isopropylmalate |  |  |
| 2-Isopropylmalate | 3-Isopropylmalate |  |  |
| 2-Isopropylmalate | 3-Isopropylmalate |  |  |
| 3-Isopropylmalate | 2-Isopropyl-3-oxosuccinate | +1 NADH |  |
| 2-Isopropyl-3-oxosuccinate | 4-Methyl-2-oxopentanoate | +1 CO_2_ | 2-Isopropyl-3-oxosuccinate |
| 4-Methyl-2-oxopentanoate  +  L-Glutamate | L-Leucine  +  2-Oxogluterate |  |  |

The initial steps in l-leucine synthesis are identical to those in l-valine synthesis. However, in the l-leucine pathway, 3-methyl-2-oxobutanoate is converted into 2-isopropylmalate, utilizing one mole of acetyl-CoA. Therefore, the redox equivalents associated with acetyl-CoA formation were also taken into account. From one mole of glucose, 2 moles of acetyl-CoA can be produced, resulting in a redox balance of 2 ATP and 4 NAD(P)H. Given that a total of four l-leucine molecules are required for surfactin synthesis, the resulting metabolic balance of redox equivalents was determined to be 12 moles of ATP and 16 moles of NAD(P)H.

3-Hydroxy fatty acid

In addition to the amino acids, the hydrophobic moiety is essential for surfactin synthesis. The redox equivalents necessary for the synthesis of 3-hydroxy-13-methyltetradecanoic acid were determined. The synthesis of this fatty acid requires two different precursors. For 3-hydroxy-13-methyltetradecanoic acid, isovaleryl-CoA, which is derived from L-leucine biosynthesis, serves as a direct precursor. The redox equivalents required for the production of 1 mole of isovaleryl-CoA were calculated to be 3 moles of ATP and 4 moles of NAD(P)H. The second precursor, malonyl-ACP, is synthesized using acetyl-CoA as the initial substrate, with direct intermediates originating from glycolysis. Table S7 outlines the detailed steps involved in one elongation cycle during the synthesis of a 3-hydroxy fatty acid.

**Table S7. Redox equivalents during the conversion of pyruvate to 3-hydroxy-13-methytetradecaonic acid.**

| **Reaction** | | **Redox equivalent** | **Carbon** |
| --- | --- | --- | --- |
| 2x Pyruvate | 2x Acetyl-CoA | +2 NADH | +2 CO_2_ |
| 2x Acetyl-CoA | 2x Malonyl-CoA | -2 ATP |  |
| 2x Malonyl-CoA | 2x Malonyl-ACP |  |  |
| Malonyl-ACP  +  Isovaleryl-CoA | 3-Keto-5-methylhexanoyl-  ACP |  |  |
| 3-Keto-5-methylhexanoyl-  ACP | 3-Hydroxy-5-methylhexanoyl-ACP | -1 NADPH |  |
| 3-Hydroxy-5-methylhexanoyl-ACP | Trans-2-methylhexanoyl-ACP |  |  |
| Trans-2-methylhexanoyl-ACP | 5-Methyhexanoyl-acyl-ACP | -1 NADPH |  |
| 5-Methyhexanoyl-acyl-ACP  +  Malonyl-ACP | 3-Keto-7-methyloctanoyl-  ACP |  |  |

From one mole of glucose, 2 moles of pyruvate can be produced, which subsequently allows for the formation of 2 moles of malonyl-ACP. One of these malonyl-ACP molecules enters the fatty acid cycle, as outlined in the referenced process. In the second elongation step, the second malonyl-ACP can also enter the cycle (as detailed in the final step of Table S7). Considering the redox equivalents produced during glycolysis (2 moles of ATP and 2 moles of NAD(P)H), the overall balance for fatty acid elongation is 0 moles of ATP and 0 moles of NAD(P)H. As previously mentioned, isovaleryl-CoA, a direct precursor, contributes a redox equivalent balance of 3 moles of ATP and 4 moles of NAD(P)H, which represents the total balance for the fatty acid synthesis
